# Best Practices for Teaching Clinicians to Use a Serious Illness Conversation Guide

**DOI:** 10.1089/pmr.2020.0066

**Published:** 2020-07-28

**Authors:** Bethany-Rose Daubman, Rachelle Bernacki, Mark Stoltenberg, Erica Wilson, Juliet Jacobsen

**Affiliations:** ^1^ Division of Palliative Care and Pediatrics, Massachusetts General Hospital, Boston, Massachusetts, USA.; ^2^ Harvard Medical School, Boston, Massachusetts, USA.; ^3^ Department of Psychosocial Oncology and Palliative Care, Dana-Farber Cancer Institute, Boston, Massachusetts, USA.; ^4^ Brigham and Women's Hospital, Boston, Massachusetts, USA.; ^5^ Ariadne Labs, Harvard School of Public Health, Brigham and Women's Hospital, Boston, Massachusetts, USA.

**Keywords:** communication guide, palliative care communication, serious illness communication

## Abstract

With the palliative care workforce shortage and changes in advance care planning reimbursement, many institutions are requesting that palliative care specialists provide serious illness communication training across their institution's workforce. Based on our experience training clinicians to use the Partners Serious Illness Conversation Guide, a structured guide to teach basic palliative care communication skills, we propose a set of best practices to help others teach use of a communication guide at their institution, including fostering a safe learning environment, explicit teaching of structured communication, and preparing cofacilitators to adapt to differing skill levels of learners.

## Introduction

Serious illness communication is a skillset that is becoming increasingly valued by both general and specialist clinicians, as well as health care institutions at large. There is increasing recognition that eliciting patients' goals in the setting of serious illness is an important component of care, and that early goals of care conversations are associated with better quality of life, improved goal-concordant care, reduced utilization of nonbeneficial medical care at the end of life, and reduced cost of care.^1–4^

With the palliative care workforce shortage expected to worsen in the coming years, and the fact that these communication skills have not typically been a part of traditional medical training, many institutions are recognizing the need to train all clinicians in at least a basic level of serious illness communication.^5^ The 2016 change in Medicare reimbursement for advance care planning (ACP) has further increased institutional interest.^6^ Whereas ACP work may have previously been “outsourced” to ACP facilitators or palliative care specialists, many institutions are now recognizing the importance of serious illness communication training across the health system, and are requesting assistance from palliative care clinicians in how to train their workforce.^7–9^

**FIG. 1.**
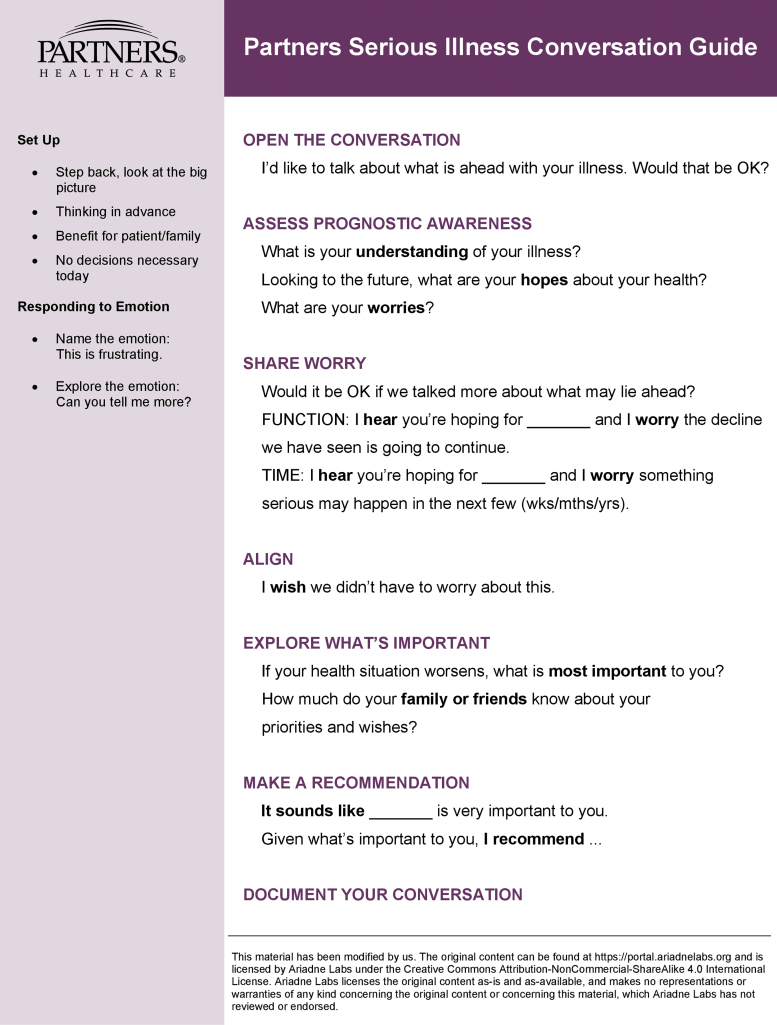
Partners Serious Illness Conversation Guide.

At our institutions (Partners Healthcare), we have adapted the Serious Illness Conversation Guide, developed by Ariadne Labs, to teach basic palliative care communication skills to interdisciplinary groups in primary care, oncology, hospital medicine, critical care, neurology, emergency medicine, and cardiology.^10,11^ In total, we have trained >2000 interprofessional clinicians across our institutions to use the adapted Partners Serious Illness Conversation Guide (henceforth referred to as the Guide, Fig. 1).

At one site, a 1000 bed academic medical center where >1250 clinicians have attended training, 2287 conversations have been documented in the ACP module by nonpalliative care clinicians from June 17, 2017 to November 16, 2019. Before the training, clinicians outside of palliative care had not been documenting serious illness conversations in the ACP module in the electronic medical record, and a brief orientation on how to document was a part of the training. The quality of documentation reveals that nonpalliative care clinicians learned a comprehensive approach to serious illness communication (Tables 1 and 2).

**Table 1.** Documentation of Each Component of the Conversation by Nonpalliative Care Trained Clinicians

Component of conversation	Documented (n* = 2287 conversations), *n (%)
Illness understanding	1939 (84.8)
Hopes	1943 (85.0)
Worries	1495 (65.4)
Prognostic information shared	1245 (54.4)
What is important	1829 (80.0)
Recommendation	1699 (74.3)

**Table 2.** Documentation of Prognostic Information Shared in 1245 Conversations Conducted by Nonpalliative Care Trained Clinicians (54.4% of Total Conversations Documented Reported That Prognostic Information Was Shared)

Prognostic information shared	Documented (n* = 1245 conversations), *n (%)
Curable	52 (4.2)
Incurable	690 (55.4)
Continued decline	558 (44.8)
Hours	13 (1.0)
Days	106 (5.8)
Weeks	247 (19.8)
Months	306 (24.6)
Years	210 (16.9)
Other	58 (4.7)

Clinicians were able to document more than one, percentages do not add up to 100.

Through these training experiences, we have developed a set of educational best practices and principles for how to effectively facilitate small group role-play using the Serious Illness Conversation Guide. Teaching communication skills using role-play is an advanced skill.^9^ Facilitators with limited prior training or experience may feel underqualified to teach colleagues and lack confidence to moderate groups effectively. Alternatively, facilitators with substantial clinical experience but limited teaching experience may sometimes attempt to teach too much information at once, overwhelming learners.

Successful preparation of facilitators from across the spectrum of skill and experience, therefore, requires clear, specific, and structured guidance on how to conduct the session. This article aims to outline how palliative care clinicians can train nonpalliative care clinicians in how to use a Serious Illness Conversation Guide using role-play.

When teaching use of the Serious Illness Conversation Guide, our sessions are generally 2.5–3 hours. The first hour is a large group session comprising five parts: (1) guided reflection on participants' own experiences with serious illness communication (10 minutes), (2) review of the research data to support serious illness conversations (15 minutes), (3) orientation to the structure of the Serious Illness Conversation Guide (15 minutes), (4) demonstration of how to use the Guide (15 minutes), and (5) opportunity to practice using the Guide in partners using a scripted drill (5 minutes). The scripted drill is done in pairs as a way for participants to practice using the language of the Guide before breaking into small groups. With one participant serving as the patient and one as the clinician, the clinicians practice the Guide by simply reading out loud an example of a serious illness conversation.

Clinicians then break into small groups for the role-play component of training (1.5 hours). Small groups generally consist of three to six clinician learners paired with one to two communication facilitators and a standardized patient. We ask the facilitators to maximize the time in small groups so that each learner has a chance to role-play and receive feedback in the 1.5-hour small group session (e.g., when there are six learners in a group, then each learner spends 15 minutes role-playing and receiving feedback during the 90-minute small group session).

## Set Up a Safe Learning Environment

### Case vignette part 1

You are a palliative care clinician tasked with teaching oncology clinicians at your hospital how to use a Serious Illness Conversation Guide. After your team demonstrates use of the Guide, learners break into small groups to practice with a standardized patient. Your group contains a senior oncologist, who has already folded up the Guide, put it in his pocket, and informed you that he needs to start clinic in 20 minutes. Next to him sits an oncology nurse practitioner who is widely known to have excellent communication skills. She mentions that she has never used this Guide but “already does basically the same thing just more naturally in conversation.”

One of the chemotherapy infusion nurses is also present, though he says he is not sure he should be practicing the Guide, as “it's beyond my scope to break bad news” and something they usually leave to the doctors. An internal medicine resident on her oncology rotation eagerly asks to go first. She is very familiar with role-plays from training in her medical school's Simulation Center. As you gather the learners in this small group, you realize how disparate their learning needs are, and feel anxious about setting the right tone as the group begins.

## Best Practices for Setting Up a Safe Learning Environment

Since practicing the Serious Illness Conversation Guide requires learner participation, the facilitator needs to establish a safe and productive learning environment.^12^ Learners are often concerned about receiving negative feedback or harsh critiques, and may arrive to the group anxious, annoyed, or disengaged.

Some common pitfalls for facilitators are as follows:

- The group refuses to do the role-play (and instead actively engages in questions and conversation about the relevance of the approach)
- Participants give critical feedback to their peers
- Time runs out and some learners do not have the opportunity for practice

The following strategies help avoid these pitfalls and enable the group to quickly develop a supportive culture that enables them to function optimally:

### Acknowledge everyone's expertise

Participants should be encouraged to learn from each other. Facilitators signal the importance of everyone's expertise through introductions (specialty, discipline, and an ice-breaker question, e.g., favorite food). It can be especially helpful to acknowledge the experience of senior clinicians who may not feel as comfortable in a learning role. For example, the facilitator might say, “You have been doing this for thirty years. It is nice to have a lot of experience in this room to help guide us.”

### Get the feelings out

After introductions, we recommend an “I hate role-play” exercise. The facilitator can lead this: “In palliative care we like to share feelings. So, let's get our feelings out about role-play. Does anybody hate role-play?” The ensuing discussion encourages learners to share (and process) feelings of vulnerability, anger, and worry.^12^ Anticipating the artificial contrived awkward set up of role-play that does not reflect one's true clinical ability decreases performance anxiety and increases participation. If feelings are not shared, they will distract participants during skills practice. Once shared, the facilitator(s) can prompt learners to discuss why role-plays are useful despite the challenges.

### Build on capabilities

Create a safe learning space by positively recognizing learners' skills. Using skills taught at VitalTalk,^13^ we begin the debrief with asking the learner, “What went well?”^14^ Then ask the group for their observations of what went well. Repeating the question cues the group that you are eliciting positive comments. If a learner responds by describing what *didn't* go well, again ask, “What did Janet do well?” Seeing this pattern and knowing facilitators will maintain safety, learners will relax and engage in the exercise.

### Monitor the time

Keeping time with a timer ensures fair allocation and sets clear expectations of when the role-play will end. Rather than leading to rigidity, this practice adds to psychological safety because the learners know that their time in the “hot seat” is limited. We strongly recommend facilitators either use a timer or if using a phone, use a timer application.

### Ensure confidentiality

Instruct the group to follow “Vegas Rules,” that is, “what happens in Vegas stay in Vegas,” that is, what happens in the role-play remains in the room and is not discussed outside this setting. Because learners are trying new language that can feel awkward or inauthentic, it is essential that they feel “free to fail.”

## Teach How to Communicate Using a Guide (Structured Communication)

### Case vignette part 2

You just finished the training session and have mixed feelings about how it went. Despite your clear explanation to the group that the goal of the session was to use the Guide, the resident eagerly started the role-play without using it, which you corrected. However, during the senior oncologist's turn, he veered off the Guide and focused on chemotherapy options, saying, “It's important to help the patient make a decision after breaking bad news.” You wonder how best to teach clinicians to say the words on the Guide and focus on values, rather than decisions.

## Best Practices for Teaching Structured Communication

The Serious Illness Conversation Guide is consistent with the education theory of task-centered instructional design, which presumes that optimal learning occurs with a focused task with readily accessible procedural or “just in time” information.^15^ The focused task makes it clear what the learner should do. The procedural information gives the learner the information they need to accomplish the task.

In the communication session, the task is to have a conversation about the patient's goals and values. The procedural information is the use of the Guide. Procedural information decreases the learner's cognitive load so that they do not get so caught up trying to remember the steps that they cannot complete the task.^16^ Because task-centered instructional design is novel, most learners lack experience with this scaffolded approach and consequently, may be resistant to it or unable to fully understand what is expected from them.

Some common pitfalls learners may encounter when practicing structured communication are as follows:

- Learners do not understand their task is to follow the Guide
- Facilitators do not provide enough structure, and fail to reorient the learner to the task
- Learners do not understand the goal of understanding patient values

The following strategies help facilitators avoid these pitfalls and facilitate a more effective role-play experience:

### Use a drill

During the didactic portion of the Serious Illness Care training, there is a demonstration of the serious illness conversation (the task) and the option for learners to participate in a drill.^13,17^ Both the demonstration and the drill offer the learner model solutions, examples of expert communication. In addition, the drill provides practice and can serve as a reference for later self-directed learning. The drill prepares the learner to practice with less scaffolding using only the Guide.

Unlike the drill, the Guide offers structure for the conversation but also requires the learner to actively direct the conversation by choosing when to allow for silence, when to respond directly to the patient, and when to proceed through the questions on the Guide. By practicing the drill first, the learner better understands the basic task of asking the questions on the Guide, and is better prepared for the more advanced task of interweaving the structure of the Guide with real-time patient responses.

### Focus on the first learner

Once in the small group role-play setting, the first learner sets the tone for the group. If the first learner understands the goal of the exercise and can model how to use the Guide effectively, the learners who follow are more likely to do the same. The converse is also true; when the first learner cannot use the Guide, others frequently “go rogue” using communication skills they already know, rather than learning new skills. For this reason, we are particularly careful to set up the first learner for success.

Somewhat paradoxically, learners who are early in training often have an easier time with the Guide. Sometimes, more senior learners see a communication cue from the patient and reflexively stray from the Guide to use their own solutions. Early learners recognize fewer cues and do not have fixed solutions, so can more flexibly adopt this new approach. When possible, ask a junior clinician to role-play first and emphasize that the goal is simply to practice the new approach of using a Conversation Guide. Set up your first learner for success through the following:

1. Make sure the learner physically has the Guide in his/her hands and understands he/she is meant to read and refer to it during the conversation.
2. Remind the learner that the goal of the exercise is to practice using the words on the Guide. This direct instruction just before the exercise helps lower the stakes for the learner and makes your expectations clear.
3. Remind the learner to actually introduce the Guide to the patient. One can suggest “I'm using a guide to help with this conversation and ensure I don't miss anything.” Although the didactic portion of the training does demonstrate how to introduce the Guide to patients, many learners still do not believe that we want them to use the Guide in the clinical encounter.
4. Instruct learners to hold the Guide with the left hand, so that they can use their left thumb to keep track of where they are in the conversation. Marking the last question with one's thumb enables the clinician to make eye contact with the patient, and then easily return to the next question in the Guide.
5. Stop big mistakes early. Remember that the rest of your learners are imprinting on this conversation. If the learner strays from the Guide asking question after question that is not on the script, the goal of the exercise becomes unclear for everyone. The further off track the learner goes, the harder it becomes to stop. If a learner is going off on a tangent, gently bring them back. “I am going to ask you to get back to the questions on the Guide. Let's start with this one.” It may be helpful to set explicit expectations with learners who are straying off the Guide. For example, if the learners feel they need to respond to something the patient has said, one or two follow-up questions or comments are acceptable before returning to the Guide, but more are not.

### Highlight the goal of eliciting values

**FIG. 2.**
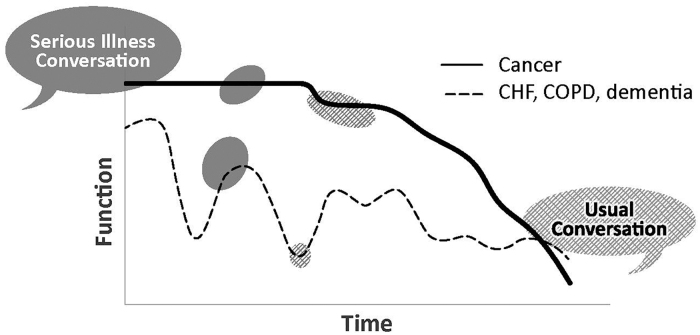
Differentiating between usual conversations and value-centered serious illness conversations.

Either through experience, observation, or teaching, most clinicians have learned to focus goals of care conversations on making decisions, and not on learning patients' goals and values. To differentiate between usual decision-centered conversations and serious illness conversations, we find the following figure helpful (Fig. 2). The figure illustrates that serious illness conversations happen early in the disease trajectory when patients are feeling well, and can, therefore, be exclusively focused on values because no specific decisions need to be made at the current time. This is juxtaposed to usual decision-centered conversations that often happen when a patient's condition has acutely worsened, where the focus is on acute medical decisions such as code status.^18,19^

We train our facilitators to reassure learners that serious illness conversations require new skills with which they may be unfamiliar, which is, in part, why the Conversation Guide was developed. Once learners have a clear understanding of how these value-centered conversations differ from usual practice, they are more likely to try the approach.

When learners do mistakenly focus on decisions, we train facilitators to quickly time out and redirect. “I am going to time out and refocus the conversation on the patient's values. This conversation is about what values will eventually inform the decision about resuscitation, but we don't have to go into that now.”

## Adapt to Learners

### Case vignette part 3

With further reflection, you realize that you were relieved when the oncology nurse practitioner used the Guide skillfully, but felt unsure whether her experience was challenging enough. In contrast, when it was the infusion nurse's turn, he noted feeling uncomfortable with the whole exercise and again emphasized that he would never be discussing prognosis within his clinical role. You wonder how to adapt the training to learners with different roles and different skill levels.

## Best Practices for Adapting to Learners

Large scale institutional trainings using the Serious Illness Care Program often consist of learners from a range of specialities and disciplines who, in turn, bring a range of clinical experience. Some learners have given us feedback that they have enjoyed training as interprofessional groups, learning from each other's role. Other learners have reported that they prefer training in role-specific groups. For example, some of our oncology physician colleagues found it helpful to share role-specific tips about disclosing prognosis. Regardless of how the role-play groups are structured, there will undoubtedly be learners with different learning goals and needs.

Some common pitfalls for working with a range of learners are as follows:

- Learners who get stuck on the prognosis
- Advanced learners who are not adequately challenged

The following strategies help facilitators manage these common pitfalls and optimize the learning experience for all participants.

### Offer choice for the prognostic disclosure

Clinician comfort with discussing prognosis varies widely depending on the clinician's role, level of experience, and the culture of his/her clinical team, group practice, or institution. The nature of the information shared also impacts clinician comfort. For example, more clinicians are comfortable sharing prognostic information about disease trajectory: “the changes we have been seeing are likely to continue,” than they are sharing information about time: “something serious could happen in the next few weeks.”^8,20^ Within the category of time, it is usually more challenging for clinicians to share specific information, “I worry your time may be as short as 1–2 months.”

Because of the widespread variation in clinician comfort discussing prognosis, we recommend giving learners a choice. Our version of the Guide offers two approaches, one for time and the other for function (Fig. 1, the Guide). Many interprofessional clinicians discuss functional prognosis routinely. For example, a speech pathologist may align with patients' hope that their swallowing abilities improve so they can eat comfortably, yet also worry that the aspiration may continue.

For clinicians who feel uncomfortable with discussing time or function, we offer a third approach: mirroring. With mirroring, the clinician reflects back the patient's hopes and worries, but does not offer additional information. For example, the clinician might say, “I hear you are hoping for a cure and that you are worried you could be a burden and even die from this illness.” We find that interprofessional clinicians often feel that it is beyond the scope of their practice to explicitly discuss prognosis, and therefore prefer mirroring. Mirroring is also aligned with the primary purpose of the Guide, to help patients reflect on and articulate their thoughts and feelings about the future.

Finally, to ease clinician discomfort with discussing prognosis, over time the language on our Guide was modified from “discuss prognosis” to “share worry.” This change was in response to clinician concerns about the difficulties of managing prognostic uncertainty or “knowing” the prognosis. In trainings, we observed that a focus on sharing worry has decreased learner anxiety and improved compliance with practicing the prognostic disclosure in the Guide.

### Challenge advanced learners

Because watching the same conversation repeatedly can be tedious, the role of the facilitator is to maintain a dynamic environment. Adding depth to the skills that are being taught allows educators to respond to the needs of experienced learners, manage learners who are resistant to the Guide or simply unable to adapt their practice habits to use this new approach, and maintain the interest of all learners. Two ways to deepen learners' skills are to (1) elaborate the questions and (2) respond to emotion.

1. Teach learners to elaborate the goals and values questions on the Guide. The Partners Guide (Fig. 1) contains three goals and values questions: What are your hopes? What are your worries? If your health were to worsen, what is most important? Any goals and values question, including these or others, can be elaborated. Elaborate for depth by asking, “Can you tell me more?” Elaborate for breadth by asking, “And what else?” This offers experienced learners flexibility and challenge.
2. Offer learners one or two skills for responding to emotion. Being able to respond to emotion is essential for competent use of the Guide. Emotion is most prevalent at the start of the conversation and after the prognostic disclosure. In fact, emotion after the prognostic disclosure is so predictable that our version of the Guide incorporates a scripted empathic response to help the clinician manage prognosis-related emotion: “I wish I had better news.”

For advanced learners, we will sometimes ask the standardized patients to increase the intensity of their emotional response. In such cases, the scripted empathic response of “I wish I had better news,” is not sufficient, and the learner has the opportunity to add a second empathic response. We have found that an expression of empathy such as, “I can't imagine how hard this is,” is easiest for most learners to practice.^21^ To set up the exercise and facilitate skill adoption, we write the second skill for responding to emotion on a white board so the learner can easily read it during the practice. If they forget to use it, we point at it.

## Conclusion

Health care institutions increasingly recognize the need to train clinicians in serious illness communication. Through our experiences using the Partners Serious Illness Conversation Guide to teach general palliative care communication skills to a wide variety of clinicians, we have distilled best practices to help others teach how to use a communication guide at their institutions. These practices include fostering a safe learning environment, explicit teaching of structured communication, and preparing cofacilitators to adapt to differing skill levels of learners.

## Abbreviation Used

ACP: advance care planning
